# Menstrual Cycle Irregularity and Metabolic Disorders: A Population-Based Prospective Study

**DOI:** 10.1371/journal.pone.0168402

**Published:** 2016-12-16

**Authors:** Marzieh Rostami Dovom, Fahimeh Ramezani Tehrani, Shirin Djalalinia, Leila Cheraghi, Samira Behboudi Gandavani, Fereidoun Azizi

**Affiliations:** 1 Reproductive Endocrinology Research Center, Research Institute for Endocrine Sciences, Shahid Beheshti University of Medical Sciences, Tehran, Iran; 2 Non_communicable Diseases Research Center, Endocrinology and Metabolism Research Institute, Tehran University of Medical Sciences, Tehran, Iran, Development of Research Technology Center, Deputy of Research and Technology, Ministry of Health and Medical Education, Tehran, Iran; 3 Department of Biostatistics and Epidemiology, Research Institute for Endocrine Sciences, Shahid Beheshti University of Medical Sciences, Tehran, Iran; 4 Endocrine Research Center, Research Institute for Endocrine Sciences, Shahid Beheshti University of Medical Sciences, Tehran, Iran; Shanghai Diabetes Institute, CHINA

## Abstract

The regularity of menstrual cycles is considered an indicator of women’s reproductive health. Previous studies with a cross-sectional design have documented the relationship between menstrual cycle irregularities, insulin-resistance and the future risks for metabolic disorders. Limited data documented by prospective studies can lead to premature conclusions regarding the relationship between menstrual cycle irregularities and other conditions influencing women’s health. The present study therefore, using a prospective design aimed to assess the risk of metabolic disorders in women with a history of irregular menstrual cycles, was based on the data gathered from the Tehran Lipid and Glucose study (TLGS) an ongoing prospective cohort study initiated in 1999. Participants of the current study were 2128 women, aged between 18–49 years, followed for 15 years. Based on their menstrual cycles, the women were divided into two groups: (i) women with regular menstrual cycles (n = 1749), and (ii) those with irregular menstrual cycles (n = 379). The proportional COX regression model was used to compare hazard ratios (HRs) between the groups for the proposed events, including diabetes mellitus (DM), pre-diabetes (pre-DM), hypertension (HTN), pre-hypertension (pre-HTN) and dyslipidemia. It was found that during a 15-year follow up, there were 123 cases of DM, 456 cases of pre-DM, 290 cases of HTN, 481 cases of pre-HTN, and 402 cases of dyslipidemia. Compared to those with regular cycles, women with irregular menstrual cycles were found to have an increased risk for DM2 (age adjusted Hazard Ratios (HRs), 2.01; 95% confidence intervals (CI:1.59–3.50), the increased risk for DM, associated with irregular cycles remained significant after the adjustment for Body Mass Index (BMI), fasting blood sugar (FBS), family history of diabetes, and parity (HRS, 1.73; 95% CI: 1.14–2.64). There was no significant difference in the increased risk for pre-DM between the groups (age adjusted HRs, 1.33, 95% CI: 1.05–1.69). However, after the adjustment of BMI, FBS and family history of pre-DM, compared to those with regular menstrual cycles, irregular menstrual cycles showed an increased risk for pre-DM (HRs, 1.33; 95% CI: 1.05–1.69). No statistically significant difference was found in the increasing risk for other proposed events between the groups demonstrating that menstrual cycle irregularities could be considered a marker of metabolic disorders and a predisposing factor of the increased risk for diabetes mellitus and pre-diabetes in women with irregular menstrual cycles.

## Introduction

The menstrual cycle is a unique physiological event in the reproductive system of female mammals which makes pregnancy possible [1,2]. The first menstrual cycle (menarche) usually occurs between12 to 15 year of age; these cycles end around the of age of 50 years, i.e. at menopause [2]. The menstrual cycle is calculated as the duration from the first day of bleeding to the beginning of the next bleed. Although a menstrual cycle is 28 days on average, it could be a little shorter or longer. In general, a normal menstrual cycle usually lasts between 21 and 35 days.

The most regular menstrual cycles in women occur during the reproductive ages (21–35 years) [3,4].

Regularity of menstrual cycles is considered an indicator of women’s reproductive health; changes in the menstrual cycle have different reasons and are often attributed to ovaries-thyroid and pituitary axis dysfunctions. It has been shown that 87% of women with irregular menstrual cycles suffer from the polycystic ovary syndrome (PCOS); long menstrual cycles or oligomenorrhea (>35 days), often seen in PCOS women, are a result of ovarian dysfunction and insulin resistance [5,6]. An increased risk for diabetes type 2 (DM 2) in women with PCOS has been reported in previous studies [7–10]. Irregular menstrual cycle, one of the components of PCOS, may be associated with DM2 and cardiovascular diseases (CVDs) [11]. Population-based studies have also indicated the increased prevalence of DM2 and CVDs in women with irregular cycles [12–14]. A longitudinal study demonstrated the improvement of ovulatory cycles in women with PCOS and reduction of their risk for cardiovascular and metabolic syndrome [15]. In addition, it seems that menstrual cycle can be a predictive factor of the necessity of screening women for metabolic disorders; some other studies however found no statistically significant association between PCOS and metabolic syndrome [16,17].

Controversies in the findings of previous studies and a lack of knowledge gained from prospective studies can lead to premature conclusions regarding the relationship between menstrual cycle irregularities and other conditions influencing women’s health highlighting the need for more prospective studies to determine the relationship between irregular menstrual cycle and metabolic disorders. Therefore, using a prospective design. This study aimed to assess the risk for metabolic disorders in women with a history of irregular menstrual cycles.

## Materials and Methods

### Study participants

This is a prospective cohort study based on data gathered from the Tehran Lipid and Glucose study (TLGS), an ongoing prospective study, initiated in 1999 as a large scale and community-based study performed on residents of district No.13 of Tehran, the capital city of Iran[18]. The age and gender distributions of the population in this region are the representative of Tehran’s population[19]. In the TLGS study, 27340 residents were recruited at baseline, of whom 15484 women were aged >15 years. The sampling and follow-up method have been reported previously [20]. For the present study, we recruited 2128 women, aged between 18 and 49 years, with no history of endocrine disorders; they were all followed at least once for menstrual disorders after base-line data collection. For categorizing of participants into two study groups, based on their regularity or irregularity of their menstrual cycles, the Wang et al. study approach was used [14]. Women whose menstrual cycle intervals (at least in 4 out of 5 cycles) were < 35 days, they were considered to have regular cycle status (Group 1). If after two years of menarche, women reported their menstrual cycles to be completely irregular (no regularity in intervals), or reported their menstrual cycle intervals (4 out of 5) were >35 days, they were considered as having irregular cycles (Group 2).

**Fig 1:**
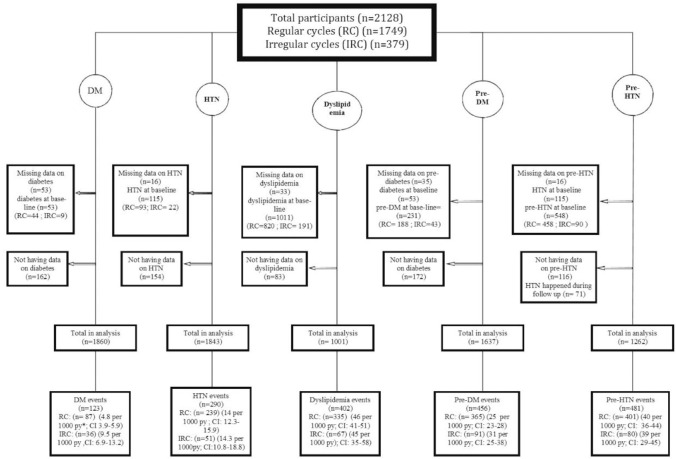
The study samples in each proposed event. Abbreviations: DM: diabetes mellitus; HTN: hypertension; Pre-DM: pre-diabetes mellitus; Pre- HTN: pre hypertension; py: per year.

The groups were compared with regard to demographic and anthropometric characteristics, and their hormonal and biochemical data. Five primary outcomes including diabetes mellitus (DM), pre-diabetes (pre-DM), hypertension (HTN), and pre-hypertension (pre-HTN) and dyslipidemia were assessed and number of participants for each outcome has been shown in Fig 1.

Participants were provided with information regarding the study methods, and their anonymity and confidentiality of their data collected during this study. Lastly, written informed consent was obtained from all participants. The ethics committee of the Research Institute for Endocrine Sciences (RIES) approved this study research proposal.

### Definition of terms and measurement of outcomes

Blood samples were taken from all participants between 7:00 and 9:00 after fasting for 12–14 hours. Fasting plasma glucose (FBS) was measured using an enzymatic colorimetric method via glucose oxidase; the standard 2-hourblood glucose test (BS-2hr) was performed only for those women who were not using any glucose lowering medications. Total cholesterol (TC) was assayed using the enzymatic colorimetric method via cholesterol esterase and cholesterol oxidase. A qualified physician measured blood pressure twice at a 10-minute interval, while the participant rested in a sitting position and the mean was reported as the participant’s BP in millimeters of mercury (mmHg). Details of information about instruments, kits, and data analysis methods have previously been reported [19].

DM2 was defined as FBS ≥ 126 mg/dl orBS-2hr ≥ 200mg/dl those women who were not using diabetes medication [21].

Hypertension (HTN) was defined as systolic blood pressure (SBP) ≥ 140 mm/Hg, or diastolic blood pressure (DBP) ≥ 90 mm/Hg for those women who were not using anti-hypertensive drugs [11]. Dyslipidemia was defined as having any of the following abnormalities in their lipid profile: (a)total cholesterol (TC) ≥6.21 mmol/L, (b)triglycerides (TG) ≥2.26 mmol/L, (c) high-density lipoprotein (HDL)1.03 mmol/L or non-HDL-c ≥4.91 (mmol/L) [22,23].Pre-diabetes(pre-DM) was diagnosed for those with blood glucose levels above the normal level, but below diabetes mellitus thresholds (100≤FBS<126) or 140≤BS-2hr<200mg/dl)[24].

Pre-hypertension (pre-HTN) was defined as120≤SBP< 140 mmHg or 80≤DBP< 90mmHg [25]. Insulin resistance (IR)was estimated using the homeostasis model assessment (HOMA) as reference standard for the measurement of insulin resistance according to the following formula: HOMA-IR = [(Fasting insulin level (mU/L) × FBS (mmol /L)]/ 22.5. In this study, IR ≥2.6 was considered as the cut off value for insulin resistance [26].

Women, minimally clothed and without shoes were measured for their weight, using a digital scale with a standard error of 100 grams. Based on the TLGS procedure, their height was measured in a standing position, without shoes, using an iron tape measure, while the shoulders were in normal alignment.

Body mass index (BMI) was calculated as weight in kilograms divided by the square of the height in meters (kg/m2). Waist circumference (WC) was measured at the umbilical site using an outstretched tape meter without pressure to body surfaces and was recorded to the nearest 0.1 cm.

A modifiable activity questionnaire (MAQ) was used for collecting data regarding physical activity patterns [27]. Participants were asked to report their levels of physical activity during the past 12 months. Leisure time physical activity was defined as performing three or more days of vigorous-intensity activity for at least 20 minutes, or five or more days of moderate-intensity activity or walking for at least 30 minutes, or five or more days of any combination of walking, moderate or vigorous-intensity activities achieving a minimum of at least 600 MET (metabolic equivalent task)-minutes per week [28,29]. In this study, based on MET/week, three groups were defined as low, moderate and high activity groups as follows: 600MET/week, over 600 MET, but < 3000MET/week and over 3000 MET/week, respectively.

### Statistical analysis

Demographic and anthropometric variables are reported as means and standard deviations (SD). The mean values and proportions of the baseline variables between the groups were compared using the t-test, and the chi-squared and Mann-Whitney tests for normal and non-normal distribution variables, respectively. The last day of follow up for data analysis was the 1^st^March 2016 to cover all proposed events.

The study period was from the beginning of the study until the time of diagnosis of each proposed event, loss to follow up or the end of the study for event analysis. If for any reason anyone refused to participate in this study or died due to any reason, they were excluded. Survival time was calculated from the beginning of the follow-up period to the date of the first incident, i.e. DM, pre-DM, HTN, pre-HTN and dyslipidemia events.

Proportional COX regression model was used to compare hazard ratios (HRs) between the groups for proposed events mentioned above. Hazard ratios (HRs) and 95% confidence intervals (CIs) were used to determine the association between potential confounders for each event and proposed events; confounders were: age, BMI, parity, FBS, BS-2hr, family history of DM, and menstrual status for DM and pre–diabetes events, which were age, BMI, systolic blood pressure, parity, family history of hypertension, and menstrual status for HTN and pre-HTN events; for dyslipidemia they were age, BMI, total cholesterol, LDL, TG, parity and menstrual status.

In this study parity has considered as a confounders for all proposed outcomes because some studies have reported that pregnancy changes hormonal status in women, and it may increase the risk of metabolic disorders [30,31]. Association between metabolic disorders and increased risk of cardio metabolic disorders has already shown[32].

The proportional hazards assumption in the COX model was graphically assessed, and it showed all proportionality assumptions to be appropriate. Kaplan-Meier survival curves were plotted for demonstrating the risk for each proposed events for the two groups. The *Log-Rank* test was used to examine the significance of the association between risks for events related to menstrual cycle status.

SPSS software (v.15; SPSS Inc., Chicago, IL, USA) and STATA software (V.12; STATA Inc., College Station, TX, USA) for Windows were used to analyze data. P-values <0.05 denoted statistical significance.

## Results

The median duration of the follow up for cohort members was 12.7 years. We documented proposed events in this population, including 123 cases of DM, 290 cases of HTN, 402 cases of dyslipidemia, 456 cases of pre-diabetes and 481 cases of pre-HTN. Incidence rates for events are displayed in Fig 1.

Participants were 2128 women. Ofwhom,1749 women had regular menstrual cycles and 379 had irregular menstrual cycles; mean (±SD) age of participants was 29.45 ± 9.16 years and 27.02 ± 8.62 years, respectively (Table 1).

**Table 1:** Comparison of baseline characteristics of participants in the groups.

	**Group 1**	**Group 2**	**P-value**
**Number of subjects**	1749	379	
**Age (yr) ^a^^,^^c^**	29.45 ± 9.16	27.02 ± 8.62	0.23
**Marital Status (%)**			
**Single**	30.5	40.4	<0.01†
**Married**	67.1	58	
**Divorced/widow**	2.3	1.6	
**Educational level (%)**			
**Illiterate**	0.9	1.3	<0.01†
**below diploma**	46.3	53	
**Diploma**	42	35.6	
**above diploma**	10.8	10	
**Body Mass Index (BMI) (kg/m2) ^a^^,^^c^**	25.29 ± 4.77	25.68± 5.27	0.09
**Waist circumference (cm) ^a^^,^^c^**	81.41 ± 11.71	82.04 ± 12.77	0.08
**Wrist circumference(cm) ^a^^,^^c^**	15.56 ± 0.95	15.60 ± 1.07	0.5
**Hip (cm) ^a^^,^^c^**	100.95 ±9.11	101.66 ± 9.75	0.16
**Waist / Hip ratio ^a^^,^^c^**	0.80 ± 0.07	0.80 ± 0.07	0.53
**FBS(mmol/l) ^a^^,^^c^**	4.89 ± 0.90	4.87 ± 0.99	0.68
**BS (mmol/l) ^a^^,^^c^**	5.92 ± 2.02	6.10 ± 1.88	0.75
**Parity(n) ^b^^,^^d^**	1(0–2)	0(0–2)	<0.001†
**Physical activity (%)**			
**High**	24.4	22.6	0.6
**Moderate**	18	20.1	
**Low**	57.6	57.4	
**HOMA-IR ^b^^,^^d^**	1.73(1.24–2.42)	1.91(1.19–2.69)	0.23
**Insulin ^b^^,^^d^**	7.95(5.70–11.03)	8.46(5.59–12.30)	0.35
**Insulin resistance (%)**	21.2	27.2	0.14
**Total Cholesterol (mmol/l) ^a^^,^^c^**	4.84 ± 1.05	4.86 ± 1.03	0.44
**LDL(mmol/l) ^a^^,^^c^**	4.84 ± 1.05	4.86 ±1.03	0.44
**HDL(mmol/l) ^a^^,^^c^**	1.15 ± 0.27	1.13 ± 0.29	0.14
**Triglycerides (mmol/l) ^a^**	1.13	1.19	<0.05†
**SBP (mmHg) ^a^^,^^c^**	108.77 ± 12.42	106.84 ±11.83	0.79
**DBP (mmHg) ^a^^,^^c^**	73.08 ± 9.19	73.22 ± 9.52	0.26
**Hypertension status**			
**Hypertension (%)**	5.3	5.9	0.66
**Pre-hypertension (%)**	26.3	24.2	
**Normal (%)**	68.3	69.9	
**Diabetes Mellitus status**			
**Diabetes Mellitus (%)**	2.6	2.4	0.94
**Pre-diabetes (%)**	10.9	11.5	
**Normal (%)**	86.5	86.1	
**Dyslipidemia (%)**	47.7	50.8	<0.05†
**Metabolic syndrome (%)**	13.9	13.9	0.53
**Ever used OCP (%)**	16.9	15.8	0.34
**Smoking (%)**	2.1	2.1	0.16
**Parental MI (%)**	13	9.2	0.04
**Parental DM (%)**	23.7	24.8	0.64
**Parental HTN (%)**	33.6	32.1	0.37

Abbreviations: DM: diabetes mellitus; HTN: hypertension; Pre-DM: pre-diabetes mellitus; Pre-HTN: pre hypertension; py: per year. OCP: oral contraceptive pill; FBS: fasting blood sugar; SBP: systolic blood pressure; DBP: diastolic blood pressure; LDL: Low density lipoprotein; HDL: high density lipoprotein; HOMA-IR’ homeostasis

^a ^Data are shown as mean ±SD

^b^ Data are shown as median

†Significant differences (P<0.05)

^c^analyzed using independent t-test for superscript

^d^Mann-Whitney test for superscript

Group1 Participants were more educated (p<0.01), were mostly married and had a higher mean parity (p<0.01), compared to Group 2.; women with irregular menstrual cycles showed a higher triglyceride levels and a higher prevalence of dyslipidemia at baseline, compared to those who had regular menstrual cycles(p<0.05).

**Fig 2:**
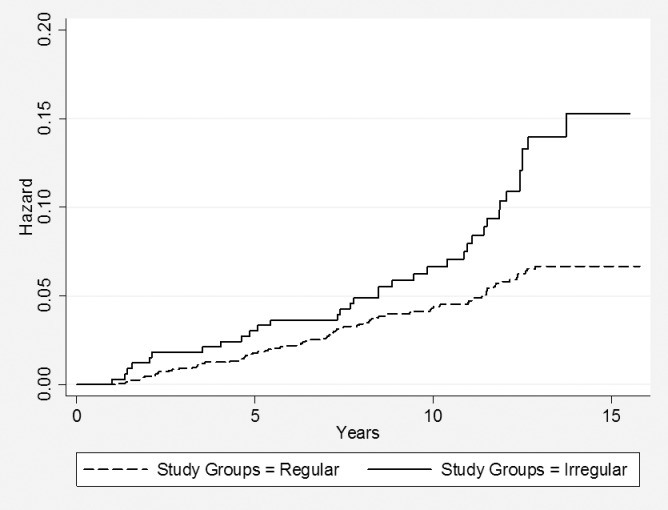
Kaplan-Meier survival curves for diabetes mellitus in the groups. P value for log rank <0.001.

**Fig 3:**
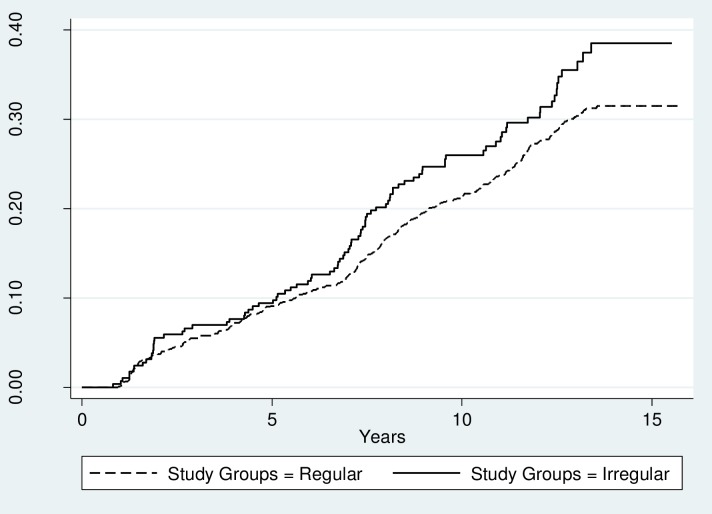
Kaplan-Meier curves for pre-diabetes mellitus in the groups. p value for log rank = 0.06.

Statistically significant differences based on the Kaplan-Miere survival curves between the study groups with regard to DM and pre-DM were observed (Figs 2 and 3).

Compared to women with regular menstrual cycles, the age adjusted hazard ratio for DM events in women with irregular menstrual cycles was 2.01, 95% CI = 1.59–3.50, and for pre-DM events this was 1.24, 95% CI = .099–1.57.

Multivariate-adjusted HRs for DM and pre-DM were 1.73, 95% CI = 1.14–2.64 and 1.33, 95% CI = 1.05–1.69, respectively. After multivariate adjustment, the hazard ratio of DM between the groups decreased, but remained significant (p<0.05) (Table 2).

**Table 2:** Multivariate adjusted hazard ratios (HRs) and 95% confidence interval for the association between the demographic and anthropometric factors and the proposed events in the groups.

**Proposed events**	**Multivariate- adjusted variables**	**HR (95% CI)**	**p-value**
**DM**			
	Age	1.02(0.99–1.05)	0.15
	BMI	1.10(1.05–1.14)	<0.001†
	FPG (mmol)	3.49(2.55–4.79)	<0.001†
	Parity	0.97(0.83–1.15)	0.85
	Family history of DM	1.73(1.18–2.54)	0.005†
	AUB (Ref: Women with regular menstrual cycles)	1.73(1.14–2.64)	0.01†
**HTN**			
	Age	1.06(1.04–1.09)	<0.001†
	BMI	1.07(1.04–1.12)	<0.001†
	SBP	1.05(1.03–1.06)	<0.001†
	Parity	0.97(0.92–1. 12)	0.64
	Family history of HTN	1.17(0.88–1.55)	0.26
	AUB(Ref: Women with regular menstrual cycles)	1.26(0.89–1.80)	0.18
**Dyslipidemia**			
	Age	1.01(1.00–1.03)	0.03†
	BMI	1.00(0.97–1.02)	0.96
	TG	3.24(2.34–4.49)	<0.001†
	LDL	5.56(3.08–10.02)	<0.001†
	Cholesterol	0.22(0.12–0.40)	<0.001†
	Parity	0.98(0.88–1.09)	0.74
	AUB(Ref: Women with regular menstrual cycles)	1.00(0.76–1.31)	0.97
**Pre-hypertension**			
	Age	1.03(1.01–1.05)	<0.001†
	BMI	1.07(1.04–1.10)	<0.001†
	SBP	1.02(1.01–1.04)	<0.001†
	Parity	1.07(0.95–1.19)	0.22
	Family history of HTN	0.84 (0.67–1.06)	0.14
	AUB(Ref: Women with regular menstrual cycles)	1.04(0.79–1.40)	0.74
**Pre-diabetes mellitus**			
	Age	1.03(1.02–1.05)	<0.001†
	BMI	1.03(1.01–1.05)	<0.001†
	FPG (mmol)	3.31(2.51–4.37)	<0.001†
	Parity	1.03(0.94–1.13)	0.48
	Family history of DM	1.52(1.24–1.87)	<0.001†
	AUB (Ref: Women with regular menstrual cycles)	1.33(1.05–1.69)	0.01†

DM: diabetes mellitus; BMI: Body mass index; FBS: fasting blood sugar; AUB: abnormal uterine bleeding; HT: hypertension; SBP: systolic blood pressure; TG: triglyceride; LDL: low density cholesterol; HOMA-IR: homeostatic model assessment -insulin resistance

*Reference group for AUB: women with regular menstrual cycles

†level of significance p <0.05

There was no statistically significant difference between HRs of pre-DM between the groups before incorporation of the adjustment model, whereas these HRs of pre-DM significantly increased in women with irregular menstrual cycles after following it.

Incidence rates of dyslipidemia, HTN, and pre-HTN did not differ significantly between the groups; older women with higher levels of BMI and SBP were mainly at risk for future hypertension (p<0.001). However, menstrual cycle status had no effects on risk of future hypertension (Table 2). It was also shown that the women with higher levels of TG, LDL, and cholesterol had higher risk for dyslipidemia, whereas menstrual cycle status had no effects on the risk of future dyslipidemia; in addition, parity did not increase the risk for proposed events.

## Discussion

Results of this study underscore the importance of the regularity of women’s menstrual cycles as a potential indicator of future metabolic disorders. To the best of our knowledge, this is the first study that has investigated the association between irregular menstrual cycles and the risks of metabolic disorders over a long follow up in a large well defined sample a cohort study of Iranian women.

We found that those women with irregular menstrual cycles had both higher triglyceride levels and higher prevalence of dyslipidemia. Moreover, compared to women with regular cycles, women with irregular menstrual cycles had increased risk for DM and pre-DM.

The role of some variables including age, BMI, and family history of diabetes in the occurrence of diabetes and hypertension are well known [33–36]; parity has also been considered as a contributing factor in some studies [37,38]; in our study we have investigated whether the risk of DM and pre-DM were attributable to the menstrual cycle irregularity, after adjustment for these variables. We found that the risk for DM and pre-DM though slightly attenuated, was still significant after adjustment for age and other confounders, suggesting that menstrual cycle irregularity is an important contributor to the risk for DM and pre-DM. Regarding incidence rates of other proposed events, no statistically significant difference was found between groups. Consistent with findings of present study, there are reports of about 50% increased risk of nonfatal myocardial infarction or fatal cardiac heart disease (CHD) in women with a positive history of irregular menstrual cycles[39]. Many investigations show a considerable association between the risk of CHD and DM with a history of irregular cycles [13,14,40].

Regarding other probable reveres outcomes, evidence shows that there is an association between insulin resistance and severe irregularity of cycles, PCOS, oligomenorrhea, and amenorrhea [41,42]. Yet, some investigations do not confirm such an association [43,44]. Controversy to our findings several coronary risk factors, including obesity [45,46], glucose intolerance [47,48], HTN [48], and lipid profile disorders have been reported among women with PCOS [40,45,46,49].

According to our findings, older women with higher levels of BMI and SBP had a higher risk for hypertension; though the menstrual cycle status however did not influence the risk for hypertension. Similarly, despite non-significant effects of menstrual cycle status, higher levels of TG, LDL, and cholesterol predisposed the women to a higher risk for dyslipidemia; also, parity did not increase the risk for any of the proposed events that could possibly be explained by the relationship between metabolic disorders and a lack of ovulation [12]. Data reveals that menstrual irregularity and PCOS, in women with metabolic abnormalities are considered as factors predisposing to CVD [39]. These outcomes rooted in changes that caused by metabolic risk factors and abnormal levels of hormones [40].

The strength of this study is its prospective nature and a large sample size. The availability of measured risk factors and biochemical measures in a subpopulation of a cohort study as a comprehensive set of data provides specific possibilities for comprehensive data analysis. As for limitations, the data on characteristics of menstrual cycle were collected via self-reports retrospectively. Results of this study should not be over generalized and existing differences between populations must be considered during interpretation of its findings.

## Conclusion

Our results s suggest that a history of irregular menstrual cycles may be an indicator of the increased risk for future DM and pre-DM, findings which may be explained by high rates of PCOS that can lead to metabolic disorders in women with irregular menstrual cycles, emphasizing the importance of screening for metabolic disorders and providing guidelines advocating healthy lifestyles, especially in women with such a history.

## Supporting Information

S1 Data SPSS data file containing variables used in analysis. (SAV)
